# SARS-CoV-2 and pulmonary embolism: who stole the platelets?

**DOI:** 10.1186/s12959-020-00229-8

**Published:** 2020-09-03

**Authors:** Michael Tran, Chirag Sheth, Rohan Bhandari, Scott J. Cameron, Deborah Hornacek

**Affiliations:** 1 Heart Vascular and Thoracic Institute, Department of Cardiovascular Medicine, Section of Vascular Medicine, Cleveland Clinic Foundation, Desk J-35, Cleveland Clinic Foundation, Cleveland, OH 44195 USA; 2 Department of Cardiovascular and Metabolic Sciences. Cleveland Clinic Lerner College of Medicine, Cleveland, OH 44195 USA

**Keywords:** Pulmonary embolism, HIT, SARS-CoV-2, COVID-19, Thrombosis, Heparin

## Abstract

**Background:**

Patients infected with SARS-CoV-2 often develop venous and arterial thrombosis. The high patient mortality is partly attributed to thrombotic events. An emerging trend is the presence of immunological phenomena including antiphospholipid antibodies which may promote thrombosis. The mechanism for these observations is not clear though many patients with SARS-CoV-2 develop thrombocytopenia.

**Case presentation:**

We describe a patient with SARS-CoV-2 pneumonitis who presented with intermediate risk pulmonary embolism (PE). Careful attention to his daily platelet count suggested the possibility of immune mediated heparin-induced thrombocytopenia (HIT) which was confirmed by laboratory testing and resolved when anticoagulation was switched to a direct thrombin inhibitor.

**Conclusions:**

Since excessive platelet activation and in situ thrombosis occur in HIT, this case underscores the need to consider that thrombocytopenia in patients with SARS-CoV-2—most of whom receive heparinoids—may be unrecognized HIT. A central role for the platelet in the etiology of thrombosis during the COVID-19 pandemic should be explored.

## Background

Critically ill patients with COVID-19 infection often have multiple abnormalities in hemostasis and thrombosis. Recent literature documents hematologic derangements including mild thrombocytopenia [1], elevated d-dimer [2], prolonged activated partial-thromboplastin time (aPTT), and disseminated intravascular coagulation (DIC) [3]. It is unclear whether these changes reflect SARS-CoV-2 infection, or an inflammatory state of acute illness. The recent medical literature reports multiple anticoagulation strategies to prevent thrombotic events in patients infected with SARS-CoV-2. Emerging reports suggest the possibility of HIT developing in SARS-CoV-2 patients receiving heparin anticoagulation [4, 5]. This case was a diagnostic dilemma since both thrombocytopenia and in situ pulmonary thrombosis are common features of SARS-CoV-2 infection [6], making less common diagnoses, such as HIT, which shares similar features, more challenging to diagnose.

## Case presentation

A 62-year-old man with type 2 diabetes mellitus presented to the emergency department (ED) with 4-day history fever, cough, and dyspnea. The patient’s vitals in the ED were as follows: Temperature 39.2 °C (102.6 °F), blood pressure 167/67 mmHg, heart rate 135 beats per min, respiratory rate 22 breaths per minute, oxygen saturation 74% on room air. The SaO_2_ improved to 96% with oxygen therapy at 10 L/minute by non-rebreather mask. Relevant laboratory data was as follows: white blood count 13.9 K/uL, platelet 412 K/uL, sodium 126 mmol/L, creatinine 0.7 mg/dL. Chest radiography showed bilateral diffuse patchy airspace opacities. There was concern for COVID-19 which was confirmed by polymerase chain reaction (PCR) for the SARS-CoV-2 amplicon. The patient developed hypoxemic respiratory failure the following day and was placed on mechanical ventilation. Over a 10-day time period, the patient was treated with investigational therapies for COVID-19 including azithromycin, hydroxychloroquine, and convalescent plasma. The anticoagulation regimen consisted of subcutaneous enoxaparin (60 mg once daily) for venous thromboembolism (VTE) prophylaxis. Intravenous unfractionated heparin (UFH) flushes were used to maintain patency of vascular access. The patient was subsequently transferred to our tertiary medical center on hospital day 12 for further management.

**Fig. 1:**
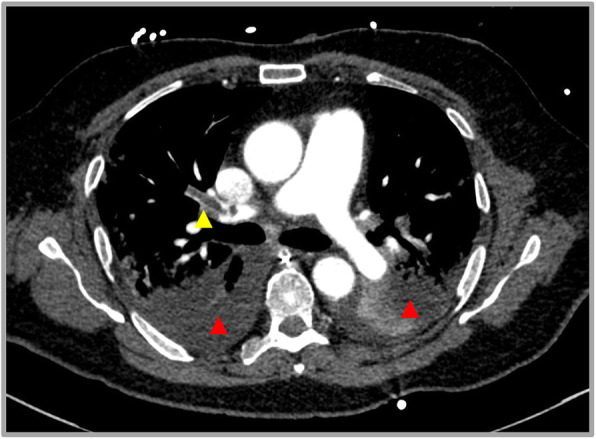
Computed tomography (CT) of chest showing a filling defect in the right upper labor pulmonary artery extending into the segmental and subsegmental pulmonary branches consistent with an acute pulmonary embolism (yellow arrowhead). Patchy infiltrates are indicated by the red arrowhead

Bedside echocardiography suggested right ventricular (RV) dilation, raising the possibility of pulmonary embolism (PE). Venous Duplex of the lower extremities was unremarkable. Chest computed tomography angiography (CTA) demonstrated right upper lobe lobar and segmental PE (Fig. 1). Relevant laboratory data included hemoglobin 11.1 g/dL, platelets 487 k/uL, creatinine 0.93 mg/dL, pro-BNP 7600 pg/mL, troponin T < 0.01 ng/mL, d-dimer 11,040 ng/mL, activated partial thromboplastin time (aPTT) 24.2 s, prothrombin time (PT) 14 s, fibrinogen 661 mg/dL. The patient had an equivocal cardiolipin IgM antibody of 15 IgG phospholipid units (MPL) since our reference range is < 12 MPL, and beta-2 glycoprotein antibodies were negative. Using the patient’s PT, d-dimer, and measured fibrinogen concentration of 661 mg/dL his DIC score is 6 which was compatible with overt DIC.

**Fig. 2:**
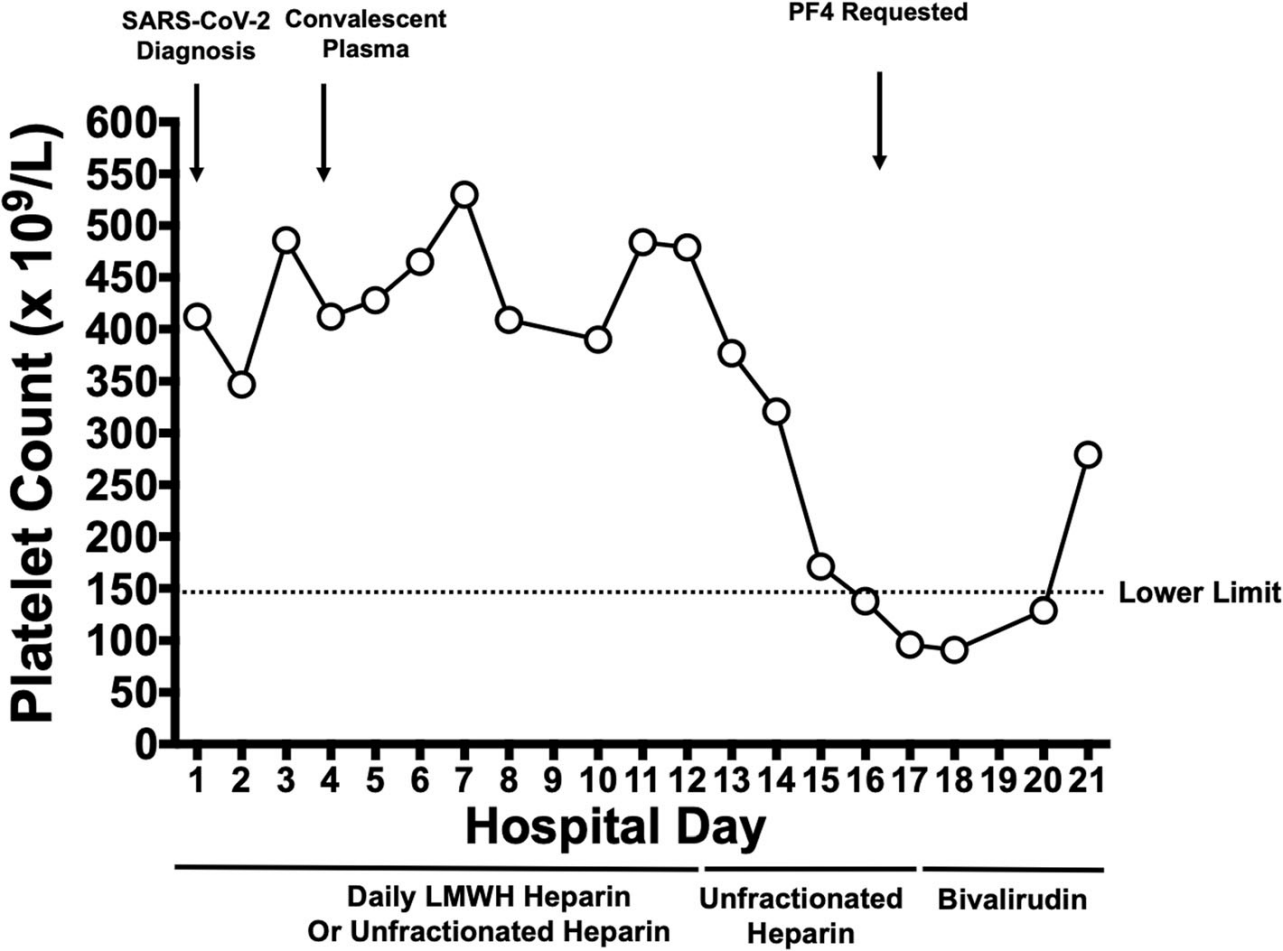
Platelet count and time points for anticoagulation administration and laboratory testing

A multidisciplinary pulmonary embolism response team (PERT) reviewed the patient’s case and recommended therapeutic anticoagulation with intravenous UFH dosed at 18 units/kg/hr and monitored by the anti-Xa chromogenic assay. The patient’s platelet count decreased from 487 k/uL to a nadir of 91 k/uL over the following 4 days, raising the concern for heparin induced thrombocytopenia (HIT) with an intermediate pretest probability by the 4Ts score of 4 (Table 1). Heparin products were exchanged for the direct thrombin inhibitor (DTI) bivalirudin dosed at 0.19 mg/kg/hr and monitored with activated partial thromboplastin time with a goal of 46 to 65 s. IgG specific anti-platelet factor 4 (PF4)-heparin enzyme linked immunosorbent assay (ELISA) was quite positive (optical density 1.08, normal value < 0.4). The heparin induced platelet aggregation (HIPA) functional assay was also positive, confirming the diagnosis of HIT. The patient’s platelet count increased to 279 k/uL three days after the discontinuation of UFH (Fig. 2). The patient responded favorably to anticoagulation with a DTI without new thrombotic complications.

**Table 1:** 4Ts Score for Heparin-Induced Thrombocytopenia

4Ts pretest probability	Score	4
Thrombocytopenia	Platelet count fall > 50% and platelet nadir > 20	2
Timing of platelet count fall	Onset after day 10 of heparin exposure	1
Thrombosis or other sequelae	No new thrombosis	0
Other causes for thrombocytopenia	Possible other causes	1

HIT is an acquired immune-mediated complication associated with UFH or LMWH administration in which platelets become haptenized [7, 8], creating a prothrombotic state that can be limb- or life-threatening due to venous or arterial thrombosis. The most common clinical manifestation of HIT is thrombocytopenia (platelet count < 150 k/uL) in 85–90% of patients between 5 and 10 days after initiating heparin products. The incidence of HIT is < 0.1–7% depending on clinical context, the heparin product used, and duration and dose of exposure [8, 9].

HIT develops when immunoglobulin G (IgG) antibodies recognize PF4-heparin complexes and activate platelet surface Fcγ receptors. This step activates platelets which degranulate and aggregate as thrombi [10]. Laboratory evaluation includes an IgG-specific anti-PF4 immunoassay which has high sensitivity but sometimes low specificity for activated platelets. Interestingly, this assay also detects non-platelet activating anti-PF4/heparin antibodies [11]. The diagnosis is supported by demonstrating IgG anti-PF4-heparin mediated platelet activation using washed platelets with functional assays such as the HIPA and serotonin release assay (SRA) [12]. The assay performance of these two offers superior sensitivity and specificity in detection of pathogenic platelet activating antibodies. As a clinicopathologic syndrome, the diagnosis of HIT depends on taking a careful history and having temporal awareness of the exposure to heparinoids [13].

## Conclusions

It is clear that patients infected with SARS-CoV-2 have unusual immunological phenomena including the presence of anti-phospholipid antibodies [14, 15]. The training of the internist is instinctually drawn to a broad differential diagnosis which is now more important than ever in the COVID-19 era. Early recognition and treatment for other immunologic phenomena such as HIT should be considered for every patient since thrombosis with thrombocytopenia often co-exist. Further study is required to determine if undiagnosed HIT contributes to the dramatically high number of thrombotic events in patients with SARS-CoV-2 infection.

## Data Availability

The datasets obtained and analyzed in the current study are available from the corresponding author on reasonable request.
